# Stable expression of a recombinant human antinucleosome antibody to investigate relationships between antibody sequence, binding properties, and pathogenicity

**DOI:** 10.1186/ar1768

**Published:** 2005-06-10

**Authors:** Lesley J Mason, Anastasia Lambrianides, Joanna D Haley, Jessica J Manson, David S Latchman, David A Isenberg, Anisur Rahman

**Affiliations:** 1 Centre for Rheumatology, Division of Medicine, University College London, UK; 2 Medical Molecular Biology Unit, Institute of Child Health, University College London, UK

## Abstract

When purified under rigorous conditions, some murine anti-double-stranded-DNA (anti-dsDNA) antibodies actually bind chromatin rather than dsDNA. This suggests that they may actually be antinucleosome antibodies that only appear to bind dsDNA when they are incompletely dissociated from nucleosomes. Experiments in murine models suggest that antibody–nucleosome complexes may play a crucial role in the pathogenesis of glomerulonephritis in systemic lupus erythematosus. Some human monoclonal anti-DNA antibodies are pathogenic when administered to mice with severe combined immunodeficiency (SCID). Our objective was to achieve stable expression of sequence-altered variants of one such antibody, B3, in Chinese hamster ovary (CHO) cells. Purified antibodies secreted by these cells were tested to investigate whether B3 is actually an antinucleosome antibody. The pathogenic effects of the antibodies were tested by implanting CHO cells secreting them into SCID mice. Purified B3 does not bind to dsDNA unless supernatant from cultured cells is added, but does bind to nucleosomes. The strength of binding to dsDNA and nucleosomes is dependent on the sequence of the light chain. Mice that received CHO cells secreting wild-type B3 developed more proteinuria and died earlier than control mice that received nonsecreting CHO cells or mice that received B3 with a single light chain mutation. However, none of the mice had histological changes or deposition of human immunoglobulin G in the kidneys. Sequence changes may alter the pathogenicity of B3, but further studies using different techniques are needed to investigate this possibility.

## Introduction

Systemic lupus erythematosus (SLE) is an autoimmune rheumatic disease of unknown aetiology, characterised by the presence of autoantibodies against a multiplicity of nuclear, cytoplasmic, and membrane antigens [1]. Autoantibodies that bind double-stranded DNA (anti-dsDNA antibodies) are present in approximately 70% of patients with SLE and are believed to play a particularly important role in lupus nephritis. These antibodies are practically specific to patients with SLE [2] and there is a correlation between increased disease activity and raised levels of anti-dsDNA antibodies in many patients [3,4]. Anti-dsDNA antibodies are found in the kidneys of patients with lupus nephritis, but not with other types of nephritis [5]. In mouse and rat models, several research groups have shown independently that some murine or human monoclonal anti-dsDNA antibodies can be deposited in the kidneys, with associated glomerulonephritis and proteinuria [6-11].

However, it has been shown in both patients and murine models that only a subset of circulating anti-DNA antibodies are deposited in the kidney and are pathogenic. Both isotype and binding properties distinguish pathogenic from nonpathogenic anti-dsDNA antibodies. Anti-dsDNA antibodies of the immunoglobulin G (IgG) isotype are believed to be the major culprits in the pathogenesis of lupus nephritis [4].

The precise binding properties of autoantibodies found in SLE are likely to affect their pathogenicity. In particular, it is increasingly recognised that some antibodies previously thought to bind dsDNA are actually antichromatin antibodies [12]. In a series of experiments, Berden and colleagues have shown that nucleosome/antinucleosome complexes in mice can cause glomerulonephritis by interacting with heparan sulphate in the glomerular basement membrane [10,11].

In previous studies, we have investigated the pathogenicity of a number of human antibodies, including the monoclonal IgG1λ antibody B3, which was derived in our laboratory from a patient with active SLE [13]. When hybridoma cells secreting B3 were implanted into mice with severe combined immunodeficiency (SCID), the antibody was shown to penetrate cells and bind to their nuclei, both in the kidney and in other organs [8]. The mice given B3 implants developed proteinuria, although histological examination of their kidneys did not show glomerulonephritis.

Sequence analysis of the heavy chain variable region (V_H_) and light chain variable region (V_L_) of B3 [14,15] showed that it possesses a number of features characteristic of IgG anti-dsDNA antibodies reported from both mice [16] and humans [17]. These include multiple somatic mutations and the presence of arginine residues at critical positions in the antigen-binding site. A computer model of the three-dimensional structure of the B3–DNA complex suggests that binding is stabilised by interaction of dsDNA with three arginines in B3, one each in the complementarity-determining region 1 (CDR1) and CDR2 of the light chain and another in CDR2 of the heavy chain [18]. One of these arginines, at position 27a in CDR1 (R27a) of the B3 λ chain, has arisen by somatic mutation from serine.

Expression and modification of murine and human anti-DNA antibodies *in vitro *has shown that removal of arginine residues often leads to a decrease in affinity for dsDNA [15,19-21]. We have expressed variant forms of B3, in which particular sequence alterations were introduced into the heavy or light chains, transiently in COS-7 cells [15,22,23]. This method was used to show that the pattern of somatic mutations in B3V_λ _is critical in determining its ability to bind dsDNA. In particular, reversion of R27a to serine (R27aS) in B3V_λ _CDR1 resulted in a significant reduction in dsDNA binding, indicating the importance of this arginine at the binding site [15,23]. When an extra arginine was introduced into CDR3 of the B3 light chain, by exchanging this CDR with that of another monoclonal human anti-DNA antibody, 33H11 [23], the resulting construct (designated B33V_λ_) conferred increased ability to bind dsDNA compared with either B3V_λ _or 33H11V_λ_.

These experiments, however, were all carried out using supernatant from COS-7 cells. The supernatants were treated with DNase, but this treatment is not sufficient to ensure that none of the antibody is present in complexes with nucleosomes. It was therefore possible that purified B3 might bind to nucleosomes or other chromatin derivatives but not to dsDNA. Expression of variants of murine anti-DNA antibodies has shown that sequence alterations that enhance binding to dsDNA do not necessarily increase pathogenicity [20]. It was therefore important for us to investigate whether the apparent effect of the R27aS sequence alteration on the ability of B3 to bind dsDNA was paralleled by an effect on pathogenicity. The amount of whole IgG produced by transient expression in COS-7 cells was too small to allow purification or for experiments on pathogenicity in SCID mice to be carried out. It was therefore necessary to establish a stable expression system for production of recombinant B3 and its variants in Chinese hamster ovary (CHO) cells.

## Materials and methods

### Assembly of 'supervectors' for expression

The 'expression supervectors' (containing both heavy chain and light chain cDNA) were adapted from the single-chain expression vectors that we previously used for our transient expression experiments [15,22,23]. The original vectors pG1D1 and pLN10 were both kindly given to us by Dr CA Kettleborough and Dr T Jones at Aeres Biomedical, Mill Hill, London.

**Figure 1:**
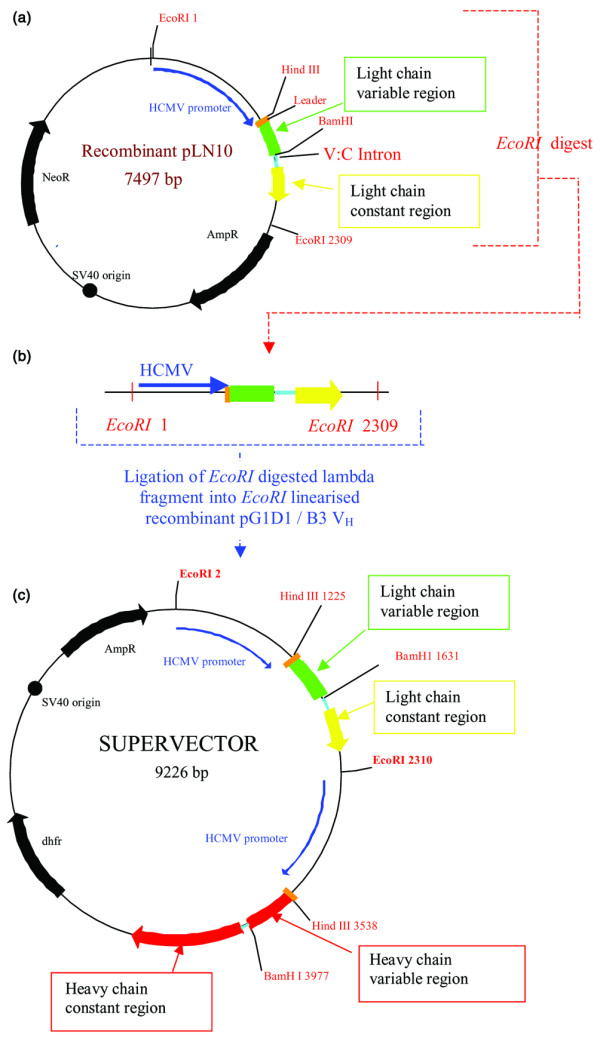
Cloning method used to construct the supervectors by combining the light chain and heavy chain expression vectors. **(a) ***Eco*RI restriction sites in recombinant light chain expression vector, pLN10, containing Vλ cloned DNA sequences. **(b) ***Eco*RI-digested light chain cassette containing human cytomegalovirus (HCMV) promoter, immunoglobulin leader sequence, light chain variable-region DNA sequence, and constant-region DNA sequence. **(c)**Ligation of light chain cassette into *Eco*RI-linearised B3V_H_/pG1D1 heavy chain vector to produce the final supervector, containing all components required to produce whole IgG1. The four supervectors were constructed in the same way, using the appropriate *Eco*RI-digested light chain fragments leading to slight variations in the overall plasmid size. SV40, simian virus 40; V:C, variable : constant.

Recombinant expression vectors – pG1D1 containing human immunoglobulin B3V_H _cDNA, and pLN10 containing human immunoglobulin V_L _cDNA – were constructed as described in detail previously [15,22,23]. V_H _sequences were ligated into pG1D1 as *Hind*III/*Bam*HI fragments, distal to an immunoglobulin leader sequence and proximal to a block of cDNA encoding the C_γ1 _constant region. The V_H _and C_γ1 _sequences are separated by an intron. Similarly, V_λ _sequences were ligated into pLN10 as *Hind*III/*Bam*HI fragments, distal to an immunoglobulin leader sequence and separated by an intron from a C_λ _sequence that lies distal to the insert. In both pLN10 and pG1D1, the inserted genes are expressed from a human cytomegalovirus (hCMV) promoter (see Fig. 1).

Four different V_L _constructs were used: these were B3V_L_, B3V_L_(R27aS), B33V_L _(which contains the sequence of B3V_L _up to the beginning of framework region 2 and the sequence of 33H11V_L _distal to that), and BUV_L _(which contains the sequence of B3V_L _up to the beginning of framework region 2 and the sequence of UK-4V_L _distal to that). 33H11 is a human monoclonal IgG anti-dsDNA antibody kindly given to us by Joachim Kalden and Thomas Winkler (Erlangen, Germany). UK-4 is a human monoclonal antiphospholipid antibody that does not bind DNA.

To produce the supervectors, an *Eco*RI fragment containing the promoter, the λ-constant-region gene, and the λ-variable-region gene (of B3 wild type or variant) from the recombinant plasmid vector pLN10 was ligated into the *Eco*RI linearised vector pG1D1/B3V_H_. This cloning scheme is shown in Fig. 1. The supervectors produced contain the genetic material necessary to express a whole heavy chain and a whole light chain.

### Stable expression of whole IgG molecules

Four different IgG-secreting lines were made. The first (designated line CHO-B3) contained cloned V_H _and V_L _sequences of the human IgG antibody B3. The others contained B3V_H _with the other three light chain constructs described above and were designated CHO-B3(R27aS), CHO-B33, and CHO-BU, respectively. The whole IgG molecules were expressed in modified CHO cells (CHO*dhfr*^-^), kindly given to us by Mrs Alison Levy (AERES Biomedical, Mill Hill, London, UK). This CHO*dhfr*^- ^cell line, DXB11, was used with kind permission from its original developer, Prof Lawrence Chasin. One allele of *dhfr *(a gene encoding dihydrofolate reductase) was deleted in DXB11; the other allele carries a missense mutation resulting in a single amino acid substitution [24].

Using electroporation (1.9 kV, 25 μF), 10 μg of recombinant supervector was transfected into 10^7 ^CHO*dhfr*^- ^cells suspended in 700 μl of PBS (pH 7.4). In each transfection experiment, a negative control sample was prepared by electroporation of the CHO*dhfr*^- ^cells in the absence of plasmid DNA. The cells were incubated overnight in nonselective growth medium (minimum essential medium, α modification (MEMα)) containing ribonucleosides and deoxyribonucleosides, 50 units/ml each of penicillin and streptomycin, and 10% ultralow-IgG FCS (all from Invitrogen, Paisley, UK)]. The cells were then grown in selective growth medium (MEMα medium without ribonucleosides and deoxyribonucleosides, 50 units/ml each of penicillin and streptomycin, and 10% ultralow-IgG FCS). The supervectors contain a functional dihydrofolate reductase gene, *dhfr*, whereas the host CHO cells do not. Consequently, only those cells that stably incorporate the supervector will survive under these selective conditions. After 10 to 14 days, foci of transfected cells were clearly visible. The foci were transferred into individual wells of a 24-well tissue-culture plate containing selective growth medium and allowed to grow until almost confluent, when the individual wells were tested for antibody production using a whole-IgG ELISA (see below). Those clones producing the highest levels of antibody were selected for expansion in selective growth medium. After expansion, these cells were submitted to methotrexate amplification either with two successive rounds of amplification at 10^-9^M and 10^-7^M methotrexate (CHO-B3 and CHO-B3(R27aS)) or with a single round of amplification at 10^-8^M methotrexate (CHO-B33 and CHO-BU).

### Production of control, stable cell line for *in vivo *experiments

A control line that had undergone the same procedures and stresses as the stable cell lines but that would not produce IgG was produced. This was achieved by transfecting the CHO*dhfr*^- ^cells with an expression vector that contained a functional *dhfr *gene but no cloned V_H _cDNA or Vλ cDNA (the 'empty vector'). Consequently, these cells were not able to express whole IgG. This control cell line was treated (i.e. selected and amplified with methotrexate) exactly as the IgG-producing cell lines were.

### Assay of antibody production in supernatant of transfected CHO*dhfr*^-^cells

The stably transfected cells were grown to near confluence in selective medium for 3 days and the supernatant was treated with DNaseI (RNase-free), 7.5 units/ml of supernatant for 1 hour at 37°C, followed by the addition of ethylenediaminetetraacetic acid (EDTA) to a final concentration of 15 mM. The supernatants were then assayed to determine the concentration of whole antibody. A viable cell count was carried out on the cells in order to calculate the level of antibody production in ng/10^6 ^cells per day. The whole-IgGλ-antibody ELISA was as described in previous papers [15,22,23].

### Affinity purification of antibody from CHO cells

The cells were transferred to Chemicon Europe Ltd (Southampton, UK) and grown in larger quantities using the selective medium described above. Human IgG was purified from the supernatant using a Protein A column and the product was analysed for purity by SDS–PAGE and quantified by spectrophotometry. Purified human IgG was sent back to our unit and the amount of antibody was checked using whole-IgG ELISA as described previously [15,22,23].

The affinity-purified antibodies were diluted in sample-enzyme-conjugate (SEC) buffer (100 mM Tris HCl, pH7; 100 mM NaCl; 0.02% Tween 20; 0.2%BSA) to a concentration of 50 μg/ml, then treated with 7.5 units/ml DNaseI at 37°C for 1 hour and then with EDTA, pH 8, to a final concentration of 15 mM to inactivate the enzyme. To investigate the effect of the DNase step, ELISA tests were also carried out on antibody diluted in SEC but not exposed to DNase.

To investigate the possible contribution of cofactors derived from cell supernatant, the same ELISA tests were carried out on antibodies diluted to a concentration of 50 μg/ml in supernatant from COS-7 cells electroporated in the absence of plasmid DNA. These supernatants contained no human IgG (confirmed by ELISA). To investigate whether nucleosomes could act as cofactors in the binding of these antibodies to dsDNA, the same ELISA tests were carried out on antibody diluted to a concentration of 50 μg/ml in SEC buffer containing nucleosomes at a range of concentrations from 1.5 μg DNA/ml to 20 μg DNA/ml (nucleosomes were prepared and quantified in terms of DNA content as described below).

### Anti-DNA ELISA

Calf thymus DNA (Sigma, Poole, UK) was further purified by phenol/chloroform extraction and sonicated to ensure reproducible coating, single-stranded DNA (ssDNA) was removed by passing the sample through a 0.45- μg Millex-HA filter (Millipore, Watford, UK), and concentration and purity were determined by spectrophotometer. This dsDNA was coated on Nunc (VWR, Lutterworth, UK) Maxisorp plates and used in an anti-DNA ELISA, as described previously [23]. Serum and ascites samples from SCID mice were diluted 1:100 in PBS/ 0.05% Tween20 (PBST) before being tested in this assay.

### Antinucleosome ELISA

Nucleosomes were prepared from Jurkat cells, grown to confluence. The cell membranes were disrupted with Dounce buffer, which causes swelling of the cells, and a fine tissue homogenizer that enables release of the nucleus without destroying it. Nucleosomes were extracted by digestion with micrococcal nuclease (final concentration 100 units/ml). Digestion was terminated by adding EDTA to a concentration of 2 mM followed by centrifugation at 600 × ***g ***for 5 min at 4°C. Aliquots were then extracted in phenol and chloroform, purified in ethanol, and run on an agarose gel to check integrity by confirming the characteristic oligonucleosome ladder pattern. The concentration of dsDNA in the nucleosome sample was approximately 1 mg/ml. This was derived by measuring the optical density at 260 nm using a spectrophotometer. This method for extraction and quantitation of nucleosomes is similar to that described by Mizzen and colleagues [25].

The nucleosome preparation was diluted 1:100 in PBS (equivalent to a concentration of 10 μg/ml dsDNA) and coated on one half of a Nunc Maxisorp plate (the test half). The other half was coated with PBS alone (the control half). The plates were washed with PBST and then blocked with 2% casein. After further washing, samples of antibody were loaded onto the plate such that each was present in a well on the test half and a corresponding well on the control half and incubated for 1 hour at 37°C. The plates were washed again with PBST. Bound antibody was detected by adding goat antihuman IgG alkaline phosphatase conjugate and incubating for 1 hour at 37°C. Substrate was added and optical density (OD) at 405 nm was read. The true OD for each sample was calculated as

OD in test well – OD in control well

to exclude effects of background nonspecific binding.

### Implantation of SCID mice with CHO cells producing recombinant IgG

Female Balb/C SCID mice were obtained from Harlan UK (Bicester, UK) at 6 weeks of age. The mice were all housed in sterile conditions on vented racks. All procedures were carried out in accordance with the Animals (Scientific Procedures) Act 1986. The mice were acclimatised for 1 week before being primed with 500 μl of pristane (2,6,10,14-tetramethylpentadecane; Sigma), injected intraperitoneally. Ten days later the mice were given implants of 1 × 10^6 ^CHO cells intraperitoneally, in 500 μl of MEMα culture medium.

Two separate experiments were conducted. In the first, five mice were given implants of CHO-B3 cells, five of CHO-B3(R27aS) cells, and four of untransfected CHO cells, and four were given only the initial pristane injection. In the repeat experiment, five mice were given implants of CHO-B3 cells, five of CHO-B3(R27aS) cells, and five of CHO cells containing the empty vector, and three received only the initial pristane injection. In the second experiment, three additional mice per group were given implants and were killed early, at days 2, 7, and 14 after implantation, to investigate human IgG levels and any pathological changes that might be transient and not seen at the end of the experiment.

Throughout the experiment, proteinuria was assessed using Albustix (Bayer Diagnostics, Newbury, Berkshire, UK). Proteinuria was scored as negative or trace that is negligible; +, 0.3 g/l; ++, 1.0 g/l; +++, 3.0 g/l; and ++++, more than 20 g/l. The mice were humanely killed either when ascites had developed to a degree that resulted in a 20% increase in body weight or when the mice became ill. Their sera, ascites fluid, and organs were collected for further analysis.

Standard solid-phase ELISAs were used to measure the concentration of human IgG antibodies, murine IgM, and murine IgG antibodies in the sera and/or ascites fluid of the mice at the end of the experiment. Serum and ascites samples were titrated from 1:20 to 1:200 000 dilution in PBST for the human IgG ELISA. Serum samples were diluted to 1:50 and 1:500 in PBST for the mouse IgG and IgM ELISA.

### Haematoxylin and eosin histological stain

Formalin-fixed, paraffin-wax-embedded kidney sections from the SCID mice were stained with H & E. The sections were then examined by a histopathologist for morphological evidence of kidney disease.

### Staining for human IgG

Formalin-fixed, paraffin-embedded kidney sections were dewaxed and endogenous peroxidase was blocked using 0.5% H_2_0_2 _in methanol for 10 to 15 min. The sections were washed in water. The sections were digested in 0.1% protease XXIV (Sigma) in distilled water adjusted to pH 7.8 with 0.1 M NaOH for 40 min at 37°C in order to expose the antigen after formalin fixation. The kidney sections were then blocked with 5% normal swine serum for 10 min. The presence of human IgG was determined by incubation with rabbit polyclonal antihuman IgG coupled to horseradish peroxidase (Dako Cytomation, Cambridgeshire, UK) for 1 hour at 37°C before development with 3,3'-diaminobenzidine.

### Enzyme histochemistry staining of neutrophils

The presence of neutrophils as seen in the H & E sections was confirmed by staining the mouse kidney and liver paraffin-wax sections for the presence of chloroacetate esterase; the neutrophils stained red and the sections were counterstained with Mayer's haematoxylin.

### Electron microscopy

When the mice were killed, a small section of each kidney was fixed in 2% glutaraldehyde/PBS and these were then embedded and processed for electron microscopy. The electron microscopic sections were then analysed and photographed by a specialist histopathologist.

## Results

### Stable expression of whole IgG molecules in CHO cells

**Figure 2:**
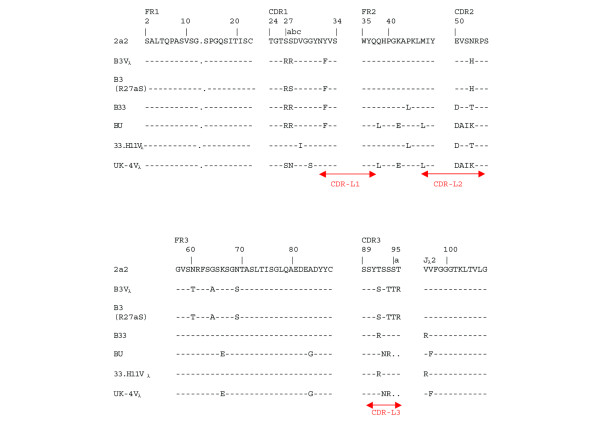
Amino acid sequences of expressed Vλ regions compared with their closest germline λ gene, 2a2. The amino acid sequences of B3V_L_, B3(R27aS)V_L_, B33V_L_, BUV_L_, 33.H11V_L_, and UK-4V_L _regions are numbered according to the system of Wu and Kabat [26]. Amino acids are indicated by their one-letter code. Dots have been inserted to facilitate the alignment. A dash indicates sequence identity with that of germline gene 2a2. The complementarity-determining regions (CDRs) and framework regions (FRs) have been defined according to the system of Wu and Kabat [26]. Antigen contact sites, as defined by MacCallum and colleagues [27], are shown by red arrows. L1, L2 and L3 are the first, second and third contact regions of the light chain respectively.

Four stable cell lines were produced, each producing IgG with the same heavy chain derived from B3, but with different light chains. These lines were named after the light chain being secreted, that is, the names were CHO-B3, CHO-B3(R27aS), CHO-B33, and CHO-BU, as described in Materials and methods. The sequences of these light chains are shown in Fig. 2. We chose these four heavy chain/light chain combinations for expression in CHO cells because previous expression in COS-7 cells had shown that they possessed a wide range of ability to bind dsDNA [23]. Thus COS-7 supernatant containing the combination B3V_H_/B33V_L _showed increased binding to dsDNA compared with the wild-type combination B3V_H_/B3V_L_. Conversely, the combination B3V_H_/B3(R27aS)V_L _showed weaker binding to dsDNA than B3V_H_/B3V_L_, and COS-7 supernatant containing B3V_H_/BUV_L_did not bind dsDNA at all.

Yields of whole IgG were different for the different constructs. After two rounds of methotrexate amplification, maximum yield for CHO-B3 was 130 ng/10^6 ^cells per day and maximum yield for CHO-B3(R27aS) was 250 ng/10^6 ^cells per day. Methotrexate amplification led to a total increase in yield of 25-to 30-fold in these lines. For CHO-B33 and CHO-BU, a single round of amplification at 10^-8^M methotrexate increased the yield of IgG to as much as 80-fold in the most productive lines. Maximum yields were 6,700 ng/10^6 ^cells per day for CHO-B33 and 148 ng/10^6^cells per day for CHO-BU. It is not clear why the CHO-B33 line should have produced so much more IgG than the others, but variation in yield depending on the construct expressed is a common finding both in this expression system and in others (discussed in [22]). The variation in yield is not relevant to the results obtained using purified antibodies, which were all tested at the same concentrations (20 to 50 μg/ml). As expected, the control CHO*dhfr*^- ^cell line, transfected with empty expression vector (i.e. containing no heavy chain or light chain variable region cDNA), produced no detectable IgG.

### Binding of affinity-purified antibodies to dsDNA

**Figure 3:**
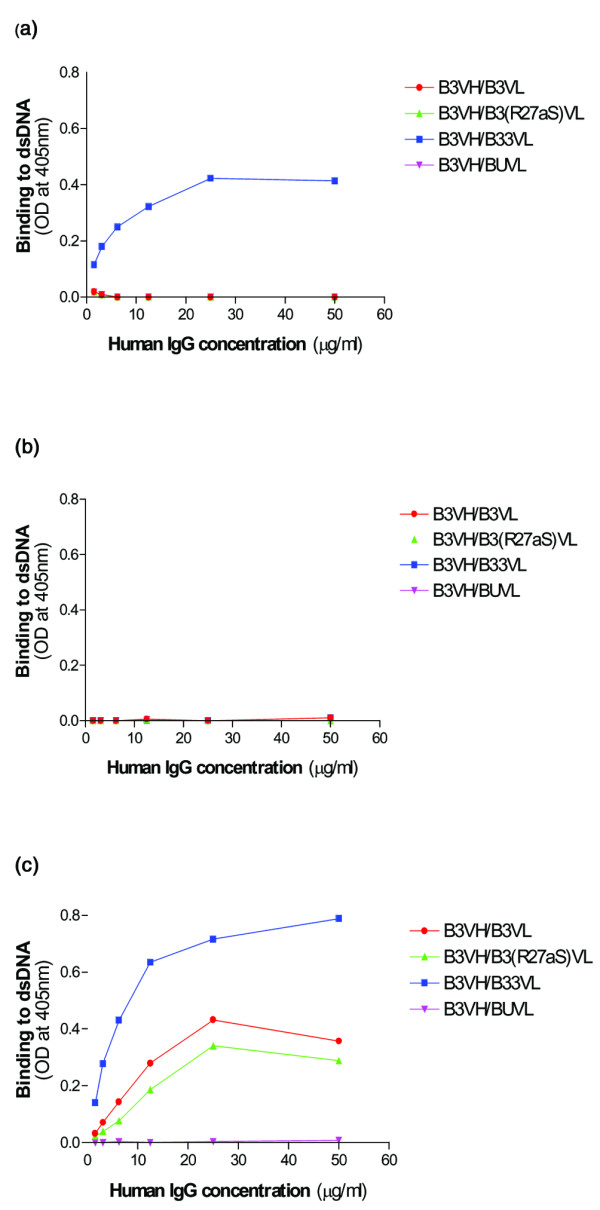
Binding of affinity-purified human IgG molecules to dsDNA. The affinity-purified human IgG antibodies were tested by ELISA for their binding to dsDNA. These experiments were carried out on two separate occasions and representative data are shown. Diluted serum from a patient with systemic lupus erythematosus was run on every plate as a positive control. The standard deviation (SD) of the standard serum optical density (OD) value between plates was ± 0.07 at a concentration of 5 IU/ml. ODs in the control wells containing no antigen were always lower than 0.068. **(a) **The dsDNA binding of the affinity-purified antibodies when diluted in sample-enzyme-conjugate (SEC) buffer but not treated with DNaseI. Only B3V_H_/B33V_L _binds to dsDNA under these conditions. The SDs were as follows: <0.121 OD units for all points on the curve B3V_H_/B33V_L _and <0.002 OD units for all points on curves B3V_H_/B3V_L_, B3V_H_/B3(R27aS)V_L_, and B3V_H_/BUV_L_. **(b) **The dsDNA binding of the affinity-purified antibodies when diluted in SEC buffer and treated with DNaseI before testing in the ELISA. The DNaseI treatment appears to have abolished the binding of the affinity-purified B3V_H_/B33V_L _to dsDNA. The SD for all points on all four curves was less than 0.002 OD units. **(c) **The dsDNA binding of the affinity-purified antibodies diluted in supernatant derived from COS-7 cells and treated with DNaseI before testing in the ELISA. The addition of the COS-7 supernatant appears to have reinstated the binding of B3V_H_/B33V_L _to dsDNA and also allows the binding of B3V_H_/B3V_L _and B3V_H_/B3(R27aS)V_L _to dsDNA. B3V_H_/BUV_L _does not bind to dsDNA. The SDs were as follows: <0.176 OD units for all points on the curve B3V_H_/B33V_L_, <0.185 OD for all points on curve B3V_H_/B3V_L_, <0.185 OD for all points on curve B3V_H_/B3(R27aS)V_L_, and <0.01 OD for all points on curve B3V_H_/BUV_L_.

Figure 3 shows binding of affinity-purified IgG from the four heavy/light combinations B3V_H_/B3V_L_, B3V_H_/B3(R27aS)V_L_, B3V_H_/B33V_L_, and B3V_H_/BUV_L _to dsDNA under different conditions. Similar results were obtained in repeated ELISAs.

When the samples are not treated with DNase after being diluted in SEC, the combination B3V_H_/B33V_L _binds dsDNA but the other three do not (Fig. 3). None of these combinations binds dsDNA at all when treated with DNase after dilution in SEC (Fig. 3b). However, when diluted in supernatant from COS-7 cells that had been electroporated in the absence of plasmid, three combinations – B3V_H_/B3V_L_, B3V_H_/B3(R27aS)V_L_, and B3V_H_/B33V_L _– all bind to dsDNA (Fig. 3c), despite treatment with DNase. The strength of binding increased in the order B3V_H_/B3(R27aS)V_L_<B3V_H_/B3V_L_< B3V_H_/B33V_L_. This was the same order seen previously by expressing these combinations transiently in COS-7 cells. The combination B3V_H_/BUV_L _does not bind dsDNA in ELISA under any conditions, which also corresponds to results obtained previously [23].

### Binding of affinity-purified antibodies to nucleosomes

**Figure 4:**
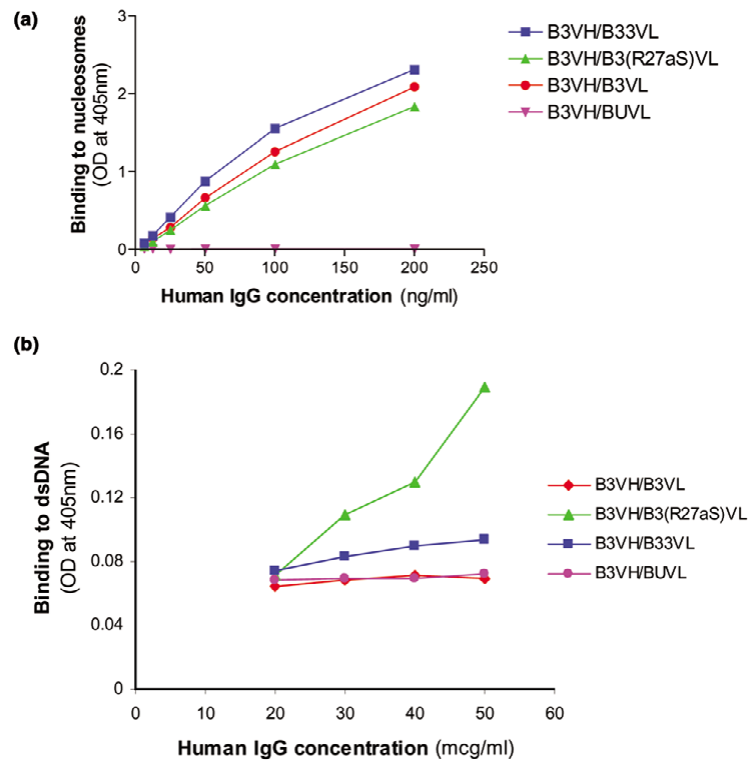
Binding of affinity-purified human IgG molecules to nucleosomes. **(a) **Binding of purified DNase-I-treated antibodies to nucleosomes in a direct ELISA. The standard deviations were as follows: <0.26 OD for all points on the curve B3V_H_/B33V_L_, <0.45 OD for all points on the curve B3V_H_/B3V_L_, <0.33 OD for all points on the curve B3V_H_/B3(R27aS)V_L_, and <0.01 OD for all points on curve B3V_H_/BUV_L_. ODs in the negative control wells were all less than 0.07. **(b)**Binding to dsDNA of purified DNase-I-treated antibodies diluted in sample-enzyme-conjugate buffer containing nucleosomes at a concentration of 10 μg dsDNA/ml. At this concentration of nucleosomes, only B3V_H_/B3(R27aS)V_L _binds to dsDNA. The same results were obtained when the experiment was repeated at a nucleosome concentration of 2.5 μg dsDNA/ml, except that the peak OD for the B3V_H_/B3(R27aS)V_L _curve was lower. At nucleosome concentrations of 1.5 μg or 20 μg dsDNA/ml, none of these heavy/light combinations bound dsDNA. The standard deviation was <0.18 OD for all points on the curve B3V_H_/B3(R27aS)V_L _and <0.01 for all the points on all the other curves. ODs in the negative control wells were all less than 0.068.

Figure 4a shows binding of the four heavy chain/light chain combinations to nucleosomes in the absence of COS-7 cell supernatant. The combinations B3V_H_/B3V_L_, B3V_H_/B3(R27aS)V_L_, and B3V_H_/B33V_L _bind nucleosomes, but B3V_H_/BUV_L _does not. The strength of binding to nucleosomes was similar for these three combinations. There is a possible trend to increased strength of binding in the order B3V_H_/B3(R27aS)V_L_<B3V_H_/B3V_L _< B3V_H_/B33V_L_, as seen in the anti-dsDNA assay, but the curves are not far enough apart to let us be certain of this trend. Binding to nucleosomes was seen at much lower concentrations than binding to dsDNA (compare Figs 3 and 4).

Since the same three combinations that bound dsDNA on the addition of COS-7 supernatant (Fig. 3c) also bind nucleosomes (Fig. 4a), we carried out experiments to test whether addition of purified nucleosomes (rather than cell supernatant) would have the same effect on the binding of these antibodies to dsDNA. We found that binding of B3V_H_/B3(R27aS)V_L _to dsDNA is reconstituted by adding purified nucleosomes at a concentration of 10 μg dsDNA/ml (Fig. 4b). This effect was also seen for this heavy/light combination at a nucleosome concentration of 2.5 μg dsDNA/ml (though the OD achieved was lower) but not at nucleosome concentrations of 1.5 μg or 20 μg dsDNA/ml. We were unable to demonstrate reconstitution of binding of purified B3V_H_/B3V_L_, B3V_H_/B33V_L_, or B3V_H_/BUV_L _to dsDNA at any of these four concentrations of nucleosomes (1.5, 2.5, 10, or 20 μg dsDNA/ml)

### Implantation of SCID mice with CHO cells producing recombinant IgG

The two lines CHO-B3 and CHO-B3(R27aS) were produced six months before the other lines. During the period when only these two lines were available, they were administered to SCID mice. The major objective of this was to investigate possible effects of the R27aS mutation on pathogenic potential of antibody B3. Table 1 shows the results of two separate experiments. The cell lines administered to the mice were the same in each experiment except that in the second case we used CHO cells transfected with empty vector as a control rather than nontransfected CHO cells. The reason for this change was that we had noted poor growth of nontransfected CHO*dhfr*^- ^cells in the mice in the first experiment and felt that using a control line that was *dhfr*^+ ^because it possessed a transfected plasmid would be a more appropriate control.

With the exception of the nontransfected CHO*dhfr*^- ^cells in experiment 1, all the implanted CHO cells grew in all the SCID mice. When the mice were killed at the end of the experiment, a few large tumour masses and many small lumps were found in the peritoneum of the mice, but no differences were observed between groups. Some of the mice had enlarged spleens or more peritoneal/ascitic fluid than others, but this occurred within all groups of mice regardless of the cells implanted.

The levels of human IgG were assayed in both the sera and ascites fluid (results not shown, as not all mice had ascites fluid), and values shown in Table 1 are from blood samples taken at the end of the experiment. In both experiments 1 and 2, the maximum levels of human IgG were found in the group given implants of the mutant CHO-B3(R27aS) cells. As expected, no human IgG was detected in any of the mice given implants of either nontransfected CHO cells or of CHO cells transfected with empty vector. The SCID mice were slightly 'leaky' at the end of experiment 2 (at age 3 to 4 months), with low levels of murine IgM found in the final blood samples of all the mice. Murine IgG was found in only one mouse, which had been implanted with CHO*dhfr*^- ^cells containing the empty vector.

**Figure 5:**
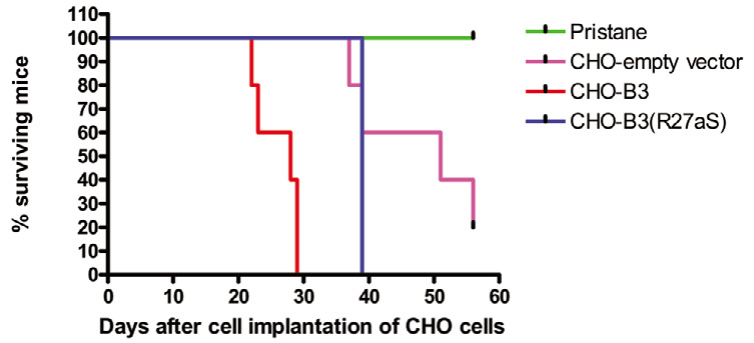
Percentage survival over time of mice given implants of CHO cells producing recombinant human IgG. This Kaplan–Meier plot shows the percentage of mice in each group that were still alive at each time point in the second experiment. The curves are significantly different by the log-rank test (*P *= 0.0001). There was reduced survival of mice in the groups that were given implants of Chinese hamster ovary (CHO) cells producing B3 and B3(R27aS), as compared with those mice in groups that were given CHO cells containing the empty vector or that were only primed with pristane. The mice were killed when they became ill; there was no evidence of ascites growth in any of these mice.

In both experiments 1 and 2, mice given implants of CHO-B3 had significantly higher levels of proteinuria, up to +++, than mice given the mutated CHO-B3(R27aS) (*P *= 0.001 and *P *= 0.05 respectively). In experiment 2, mice given implants of CHO-B3 had significantly higher proteinuria than those given CHO*dhfr*^- ^containing the empty vector (*P *= 0.001), although these mice did have some proteinuria in the range of trace to +/++. The full results for proteinuria measurement and significance tests using ANOVA and Bonferroni correction in both experiments are shown in Table 1. In both experiments, the mice that were implanted with CHO-B3 became ill and died earlier than mice in either the group that received CHO-B3(R27aS) or the control groups (Table 1 and Fig. 5). Figure 5 is a set of Kaplan–Meier curves showing a significant difference in survival between the groups (*P *= 0.0001 by log-rank test). It is particularly striking that the life expectancy of mice implanted with CHO cells containing empty vector was almost twice that of mice implanted with CHO-B3.

Despite the significant levels of proteinuria that we observed in the mice implanted with CHO-B3, H & E staining of kidney sections showed no evidence of 'lupus-like' morphology in any of the groups of mice. Staining with rabbit antihuman IgG coupled to horseradish peroxidase developed with 3,3'-diaminobenzidine also failed to detect any deposition of human antibody in kidneys from any of the mice. There was evidence of non-SLE-related pathology, namely, neutrophil infiltration of kidney glomeruli and of the liver, and liver necrosis. Such pathology occurred in all the groups of mice implanted with either CHO-B3, CHO-B3(R27aS), or CHO*dhfr*^- ^containing empty vector and was most marked in the CHO*dhfr*^- ^and empty-vector groups. This may have been due to the fact that these mice were killed later, because they were less ill. Mice killed at day 2, 7, or 14 showed consistently less pathology, with immature neutrophil infiltration, compared to mice implanted with the same cells but kept alive till the end of the experiment. Electron microscopy revealed very limited morphological changes: mesangial cell interposition, splitting of the basement membrane, and some microvillus transformation. However, these changes were present in all groups (CHO-B3, CHO-B3(R27aS), and CHO*dhfr*^- ^containing empty vector). In all groups, the foot processes were normal, and there was no thickening of the basement membrane and no evidence of immune deposits.

## Discussion

We have successfully developed a stable expression system to produce recombinant human anti-DNA antibodies. This methodology allows the investigation, both *in vitro *and *in vivo*, of the functional effects of sequence alterations in such antibodies. Even after methotrexate amplification, the expression of these antibodies by the CHO cells is low, in comparison with the amounts produced by hybridoma cells, but use of a commercial system allowed us to obtain milligram yields of purified recombinant antibody.

One interpretation of the results obtained from ELISA tests using the purified antibodies is that the wild-type antibody, B3V_H_/B3V_L_, shows binding to dsDNA strong enough to be detected by ELISA only when the antibody is complexed with some component present in supernatant of electroporated COS-7 cells. This complex dissociates when the antibody is affinity-purified, so binding is lost. When supernatant is added, the ability to bind dsDNA is restored. The same is true of B3V_H_/B3(R27aS)V_L_, but binding is weaker. The combination B3V_H_/B33V_L _binds more strongly to this component from supernatant, so that the complex does not dissociate fully during affinity purification. Thus, affinity-purified B3V_H_/B33V_L _diluted in SEC buffer binds dsDNA. That this binding is lost on treatment with DNase suggests that the component complexed with B3V_H_/B33V_L _is a bridging nucleoprotein of some kind, which is essential for binding of this antibody to dsDNA. If the complexed component were dsDNA alone, then digestion with DNaseI would increase binding to dsDNA on the plate rather than decreasing it.

This interpretation of results obtained using human antibodies is very similar to the arguments of Kramers and colleagues [10] and Guth and colleagues [12], following their experiments using murine antibodies. Kramers and colleagues showed that purification of monoclonal murine antibodies from hybridoma supernatant using DNase and high-salt conditions before loading on a protein A column was necessary to produce noncomplexed antibodies. These antibodies would bind to nucleosomes but not dsDNA, whereas if high salt and DNase were not used, the antibodies remained complexed to nucleosomes and would bind dsDNA. Guth and colleagues obtained similar results using antibodies 3H9 and SN5-18, derived from two different autoimmune mouse strains. Both antibodies bound chromatin, but not histones or dsDNA, when highly purified, whereas unpurified hybridoma supernatant or standard protein G preparation of 3H9 did bind dsDNA. Supernatant or protein G preparation of SN5-18 bound histones. When cell culture supernatant from SP2/0 cells was added to highly purified 3H9, ability to bind dsDNA was restored. The conclusion was that incomplete purification of such antibodies can lead them to appear to bind dsDNA, whereas the true antigen is a complex of dsDNA, histone 2A, and histone 2B.

It is important to note, however, that there is another possible interpretation of our results. DNase treatment may well alter DNA in solution in such a way that it acts as a competitive inhibitor of binding of antibody to dsDNA on the plate. When DNaseI is added to an incompletely dissociated complex of dsDNA with B3V_H_/B33V_L_, it may generate small fragments of DNA (perhaps ssDNA). These fragments may remain associated with the antibody or may gain access to the combining site and act as efficient competitors of binding to dsDNA. The supernatants of the dying COS-7 cells could release other nucleases that purge the combining site more effectively, thus renewing binding. However, this theory does not explain why B3V_H_/B3V_L _and B3V_H_/B3(R27aS)V_L _do not bind dsDNA in the absence of COS-7 supernatants even in the absence of DNaseI, but will bind it when these supernatants are added.

During electroporation of COS-7 cells, approximately 80% of the cells are believed to die. The supernatant of those cells is therefore rich in debris from dying cells. Nucleosomes are part of this material and contain DNA. Nucleosomes might therefore be the cofactor from the COS-7 supernatant that binds the expressed antibodies and enables them to bind dsDNA in ELISA. If this were true, one would expect the purified DNase-treated antibodies to be able to bind nucleosomes. The results shown in Fig. 4a confirm this. The combinations B3V_H_/B3V_L_, B3V_H_/B3(R27aS)V_L_, and B3V_H_/B33V_L _bind nucleosomes, without requiring the addition of cell supernatant. Guth and colleagues [12] showed that arginine-to-serine mutations in V_H_CDR3 of SN5-18 ablate binding to chromatin. The single arginine-to-serine mutation in B3(R27aS)V_L _did not ablate binding to nucleosomes but may have reduced it slightly. Replacement of CDR2 and CDR3 of B3V_L _by those of 33H11 or UK-4 gave different effects on binding to nucleosomes, even though these three light chains are derived from the same germline gene and differ only at positions of somatic mutations (see Fig. 2). In particular, the combination B3V_H_/BUV_L _does not bind nucleosomes at all.

It was puzzling that addition of purified nucleosomes could reconstitute the binding of B3V_H_/B3(R27aS)V_L _to dsDNA but not that of combinations B3V_H_/B3V_L _or B3V_H_/B33V_L_, which bind nucleosomes as well as B3V_H_/B3(R27aS)V_L _in direct ELISA. It is clear from the experiments with B3V_H_/B3(R27aS)V_L _that the concentration of nucleosomes is critical to their ability to act as a cofactor in the binding of this antibody to dsDNA. This may well also be true of the other two combinations. Perhaps some non-nucleosome component of the supernatant, such as a nuclease that removes a competitive inhibitor from the binding site, is playing a role in promoting the binding of B3V_H_/B3V_L _and B3V_H_/B33V_L _to dsDNA. Alternatively, in order to bind dsDNA perhaps these two heavy/light combinations require the presence of some nucleoprotein cofactor that is not found in our nucleosome preparation.

We postulate that the arginines at positions 27a and 92 of B33V_L _both interact with the dsDNA component of the nucleosome, as predicted by the previous computer model [23], but it seems likely that other sequence motifs on the antibody interact with the histone component to enhance antibody/nucleosome binding. These motifs are not likely to be arginine residues, as histones are positively charged.

A number of research groups have previously described stable expression of murine anti-DNA antibodies from cloned cDNA [19,20,28]. In most cases, expression was achieved using heavy chain-loss variants, which are hybridoma cells that have lost the ability to secrete heavy chains. By transfecting such variants with expression vectors encoding various different heavy chains, Radic and colleagues [19], Katz and colleagues [20], and Pewzner-Jung and colleagues [28] were all able to demonstrate that altering the numbers of arginines in CDRs of the heavy chains altered the ability of murine monoclonal antibodies to bind DNA. Of these research groups, only Katz and colleagues [20] went on to test the ability of the altered antibodies to cause pathogenic changes in mice. They produced antibodies based on the murine monoclonal anti-DNA antibody R4A. All these antibodies had the light chain of R4A, but the heavy chains were variants of the R4A V_H _sequence. They found that the antibody with strongest binding to dsDNA did not have more CDR arginines than wild-type R4A V_H_. This antibody actually showed less glomerular binding but more tubular binding to mouse kidneys *in vivo *than the wild-type R4A.

Only one research group has previously reported stable expression of whole human anti-dsDNA molecules (as opposed to Fab or single chain Fv fragments) *in vitro*. Li and colleagues [21] expressed the variable region sequences of the human IgA anti-DNA antibody 412.67 in F3B6 human/mouse heteromyeloma cells. The products were whole IgG molecules, since the expression vectors contained γ, rather than α, constant regions. In an elegant series of experiments, this group showed that reversion of two arginines in 412.67 V_H _CDR3 totally removed the ability to bind ssDNA or dsDNA. All somatic mutations outside V_H _CDR3 in either V_H _or V_L _of this antibody, however, could be reverted with no effect on DNA binding. No data were presented regarding the effect of these sequence changes on pathogenicity, and it is not known whether the original IgA antibody 412.67 is pathogenic in mice.

The stable expression of antibody B3 described here, therefore, is only the second report of stable expression of a whole human anti-dsDNA antibody *in vitro*. Expression of B3 represents the first opportunity to allow testing of the effects of sequence alterations on the pathogenicity of a human antibody already known to cause proteinuria in SCID mice.

The major finding from the experiments in the SCID mice was the marked and reproducible differences between outcomes in the mice given implants of different CHO lines. Mice given CHO-B3 died earlier and developed more proteinuria than those given CHO-B3(R27aS). Both these groups of mice developed more proteinuria and died earlier than mice in the control groups, given implants of CHO cells that did not produce IgG.

The difference in outcomes between the CHO-B3 and CHO-B3(R27aS) groups is not likely to be due to differences in tumour load or antibody expression between these groups. Serum human IgG levels were higher in the CHO-B3(R27aS) group (Table 1). These mice were therefore exposed to more human IgG but still lived longer than the CHO-B3 group. This suggests that the earlier deaths of mice in the CHO-B3 group were due either to the difference in the antibody sequence or to some direct effect of the cell line that is not so evident with the CHO-B3(R27aS) line.

It seems unlikely that the effects seen were simply due to the presence of CHO cells in SCID mice, since we used two sets of nonsecreting CHO lines as control groups. Although nontransfected CHO*dhfr*^- ^cells used in experiment 1 did not grow well *in vivo*, this did not apply to the cells transfected with empty vector in experiment 2. Although some of these mice developed proteinuria, this occurred much later, and to a lesser degree, than in the mice who received CHO-B3 or CHO-B3(R27aS). We note that intraperitoneal introduction of pristane causes inflammation, which could lead to apoptosis, necrosis, and release of nucleosomes. Since the degree of inflammation will be different in different mice, this could lead to variability in the results within groups of animals exposed to the same antibody.

The main reason to question whether the expressed antibodies actually caused the proteinuria and early death in the CHO-B3 and CHO-B3(R27aS) groups is the lack of evidence of deposition of the antibodies in the kidneys of the mice. One reason for this may be that the level of human IgG measured in the sera was very low in both the CHO-B3 and CHO-B3(R27aS) groups. In comparison, the serum levels of human IgG that were previously observed in our experiments implanting human anti-DNA hybridoma cells (including hybridoma secreting B3) into SCID mice were 1,000 times as high [8,9]. However, the level of antibody production by the transfected CHO cells *in vivo *is consistent with the level of their *in vitro *production (shown in Table 1), which is also much lower than that seen in hybridoma cells *in vitro*. Secretion of human IgG does not appear to be transient in this system, since additional mice killed earlier, at either 2, 7, or 14 days after implantation, had lower serum human IgG levels than those killed later (data not shown).

It is intriguing that, despite these low levels of circulating human IgG, the level of proteinuria observed here with concentrations of human recombinant B3 amounting to nanograms per millilitre, is the same as that observed in SCID mice implanted with hybridoma cells producing B3 [8]. This finding was reproducible and occurred even though we could not demonstrate deposition of the antibody in either the kidney or nuclei of other tissues.

Is it feasible that low levels of human anti-DNA antibodies, as secreted by the CHO cells in our experiments, are sufficient to cause the clinical outcomes of proteinuria and early death but insufficient to cause pathology observable by histological change? It appears that despite using SCID mice, the system was complicated by pathology resulting from an innate immune response, in the form of neutrophil infiltration, directed against the CHO cells. This infiltration was seen in all groups of mice implanted with CHO cells, including the nonsecreting controls. It is possible that this response may have led to up-regulation of molecules such as α-actinin, which have been postulated to be renal targets for pathogenic anti-DNA antibodies [29,30]. This could make the kidneys of these mice more susceptible to the effects of small amounts of such antibodies.

These results suggest that, although these expressed antibodies may exert pathogenic effects in mice, the limitations of our system using CHO cells in SCID mice do not allow us to be confident about this. Alternative approaches include intravenous injection of purified antibody [31], but this would only enable demonstration of renal deposition and not pathogenicity, unless repeated injections were given over a period of several days to weeks. This would require far greater quantities of antibodies than we have at present. One might also attempt to modify the expression vectors such that they could be introduced into embryos to create mice transgenic for human B3 V_H _and V_L_. However, previous experiments with murine anti-dsDNA transgenes raise the possibility that cells expressing such transgenes might be removed by deletion or receptor editing [32]. Lastly, one might apply purified antibodies to cultured renal cell lines, such as the temperature-sensitive podocyte line described by Saleem and colleagues [33] or mesangial cell lines used by Putterman and colleagues [30].

## Conclusion

In conclusion, we have established a system for the stable expression and purification of human IgG autoantibodies in CHO cells. This system was used to show that the antibody B3 is probably an antinucleosome antibody, which does not bind dsDNA after purification. Changing the sequence of B3 light chain alters binding to nucleosomes. Further experiments are necessary to investigate the effects of these changes on pathogenicity of the antibodies.

## Abbreviations

bp = base pairs; BSA = bovine serum albumin; CDR = complementarity-determining region; CHO = Chinese hamster ovary; dsDNA = double-stranded DNA; EDTA = ethylenediaminetetraacetic acid; ELISA = enzyme-linked immunosorbent assay; FCS = fetal calf serum; H & E = haematoxylin and eosin; IgG = immunoglobulin G; MEM α = minimum essential medium, α modification; OD = optical density; PBS = phosphate-buffered saline; PBST = PBS/0.05% Tween20; SCID = severe combined immunodeficiency; SEC = sample-enzyme-conjugate; SLE = systemic lupus erythematosus; ssDNA = single-stranded DNA.

## Competing interests

The author(s) declare that they have no competing interests.

## Authors' contributions

LM and AL contributed equally to this work. LM made two of the stably transfected lines and designed and carried out the experiments in SCID mice. LM and AL carried out the studies on binding properties of the purified antibodies. JH made two of the stably transfected lines and established the method for producing these lines. JM produced the nucleosomes and established the antinucleosome ELISA. DL and DI participated in the design and coordination of this project. AR conceived the study, participated in the design and coordination, and wrote the final paper. All the authors participated in the redrafting and preparation of the final version of the paper.

## Figures and Tables

**Table 1:** Summary of results from two separate experiments implanting CHO cells into SCID mice

Implanted cells	Mean human IgG in sera (ng/ml) (no. of mice/group)	Terminal proteinuria (range for group)	Mean proteinuria (estimated^a ^g/l)	Mean day of death
Experiment 1				
CHO-B3	<16 (3/5), 0 (2/5)	++/+++ to +++	2.4	20
CHO-B3(R27aS)	260 (2/5), <16 (3/5)	+ to ++	0.65	24
Nontransfected CHO only	0	Trace to trace/+	0.2	34^b,c^
Pristane only	0	Trace/+	0.2	34^b^
Experiment 2				
CHO-B3	105 (4/5), NR (1/5)	++ to +++	2.4	26
CHO-B3(R27aS)	420 (5/5)	+ to ++/+++	0.99	39
CHO with empty vector	0	Trace to +/++	0.38	48
Pristane only	0	Trace/+	0.2	56^b^

^a^Proteinuria was assessed using Albustix, which give a semiquantitative measure based on colour change. Based on values provided with the Albustix, in order to calculate the estimated proteinuria in g/l we assumed that trace = 0.1 g/l, trace/+ = 0.2 g/l, +/++ = 0.65 g/l, and ++/+++ = 2.0 g/l. The proteinuria data in both experiments were found to be normally distributed using the Kolmogorov–Smirnov test and compared using a one-way analysis of variance (ANOVA) followed by Bonferroni's multiple comparison test. In experiment 1, the ANOVA showed that there was a significant difference between the mean proteinuria values of all the groups (*P *< 0.0001). The Bonferroni post test indicated that there was a significant difference between mice implanted with CHO-B3 and CHO-B3(R27aS) (*P *< 0.001), nontransfected CHO*dhfr*^- ^cells (*P *< 0.001), and pristane only (*P *< 0.001). In experiment 2, the ANOVA showed that there was a significant difference between the mean proteinuria values of all the groups (*P *< 0.0003). The Bonferroni post test indicated that there was a significant difference between mice implanted with CHO-B3 and CHO-B3(R27aS) (*P *< 0.05), CHO cells containing the empty vector (*P *< 0.001), and pristane only (*P *< 0.01). ^b^Mice still healthy at the end of the experiment. ^c^These cells did not survive/grow in the mice. CHO, Chinese hamster ovary; NR, not recorded; SCID, severe combined immunodeficiency.
